# Structure of the poly-C9 component of the complement membrane attack complex

**DOI:** 10.1038/ncomms10588

**Published:** 2016-02-04

**Authors:** Natalya V. Dudkina, Bradley A. Spicer, Cyril F. Reboul, Paul J. Conroy, Natalya Lukoyanova, Hans Elmlund, Ruby H. P. Law, Susan M. Ekkel, Stephanie C. Kondos, Robert J. A. Goode, Georg Ramm, James C. Whisstock, Helen R. Saibil, Michelle A. Dunstone

**Affiliations:** 1 Department of Crystallography, Institute of Structural and Molecular Biology, Birkbeck College, London WC1E 7HX, UK; 2 ARC Centre of Excellence in Advanced Molecular Imaging, Clayton Campus, Monash University, Melbourne, Victoria 3800, Australia; 3 Department of Biochemistry and Molecular Biology, Biomedicine Discovery Institute, Clayton Campus, Monash University, Melbourne, Victoria 3800, Australia; 4 Department of Microbiology, Biomedicine Discovery Institute, Clayton Campus, Monash University, Melbourne, 3800 Victoria, Australia

## Abstract

The membrane attack complex (MAC)/perforin-like protein complement component 9 (C9) is the major component of the MAC, a multi-protein complex that forms pores in the membrane of target pathogens. In contrast to homologous proteins such as perforin and the cholesterol-dependent cytolysins (CDCs), all of which require the membrane for oligomerisation, C9 assembles directly onto the nascent MAC from solution. However, the molecular mechanism of MAC assembly remains to be understood. Here we present the 8 Å cryo-EM structure of a soluble form of the poly-C9 component of the MAC. These data reveal a 22-fold symmetrical arrangement of C9 molecules that yield an 88-strand pore-forming β-barrel. The N-terminal thrombospondin-1 (TSP1) domain forms an unexpectedly extensive part of the oligomerisation interface, thus likely facilitating solution-based assembly. These TSP1 interactions may also explain how additional C9 subunits can be recruited to the growing MAC subsequent to membrane insertion.

## Abstract (web summary)

The membrane attack complex is a heteromeric assembly of complement proteins where multiple copies of C9 are recruited by the C5b678 complex to form lytic pores in pathogen membranes. Here the authors present the structure of a soluble pore-like form of the C9 component that reveals details of the oligomerization interfaces.

## Graphical abstract

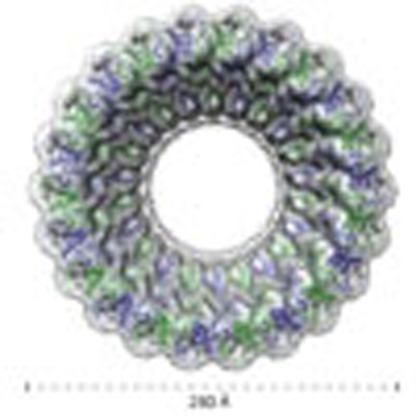

Paul Ehrlich originally characterized the haemolytic properties of human blood over 100 years ago^1^. Subsequent work revealed that the terminal ‘membrane attack complex' (MAC) portion of complement represents the lytic, pore-forming part of the system^2^,^3^. This structure is responsible for eliminating Gram-negative bacteria and other pathogens.

The MAC comprises seven components: C5b, C6, C7, C8 (a heterotrimer composed of C8α, C8β and C8γ) and multiple copies of C9 (Supplementary Fig. 1). *In vitro* studies reveal that multiple C9 subunits are recruited to the C5b678 complex, whereupon it self-assembles to form large, ring-shaped pores with a lumen over 100 Å in diameter embedded in the membrane of target cells^4^. C9 can also be induced to form poly-C9, pore-like structures in solution that closely resemble the MAC pore^5^. C6, C7, C8α, C8β and C9 all belong to the MAC/perforin-like (MACPF)/CDC superfamily^6^,^7^ and include a common set of four core domains; a N-terminal thrombospondin-1 (TSP1) domain followed by a low-density lipoprotein receptor-associated (LDLRA) domain, a MACPF domain and an epidermal growth factor (EGF) domain (Supplementary Fig. 1).

Much of our understanding of the MACPF/CDC superfamily comes from studying CDCs^8^,^9^,^10^. Briefly, soluble CDC monomers bind to and then oligomerise on the membrane surface to form a prepore intermediate^10^,^11^. Next the assembly undergoes a concerted conformational change that involves significant opening and untwisting of a central, four-stranded β-sheet. This event permits two helical regions (termed transmembrane hairpins TMH1 and TMH2) to unravel and insert into the membrane as amphipathic β-hairpins (Supplementary Fig. 2).

Studies on the MAC have revealed mechanistic distinctions from other family members. For example, perforin, pleurotolysin and CDCs bind to membrane lipids or membrane-associated proteins via ancillary domains before oligomerisation^9^,^12^,^13^. In contrast, C9 does not contain any obvious membrane-binding domain. Thus even when the nascent MAC (C5b678) is associated with the target cell, the assembly process must include the recruitment of C9 from solution (that is, from plasma, Supplementary Fig. 2b). Consistent with this, a soluble form of the MAC can also assemble independently of the membrane and be detected in blood plasma (Supplementary Fig. 2b).

To understand the mechanism of MAC assembly, we determined the sub-nanometer resolution single-particle EM structure of C9 in a polymerized pore-like form. These data reveal the unexpected finding that the TSP1 domain forms a significant portion of the interface between interacting C9 monomers. This finding may explain why the MAC, in contrast to related molecules such as perforin and the CDCs, is able to assemble from monomers directly recruited from the soluble phase. The additional interactions mediated by the TSP1 domain may also explain previous observations^14^, where C9 monomers are recruited to a MAC that has already entered the target cell membrane.

## Results

### The structure of poly-C9

**Figure 1:**
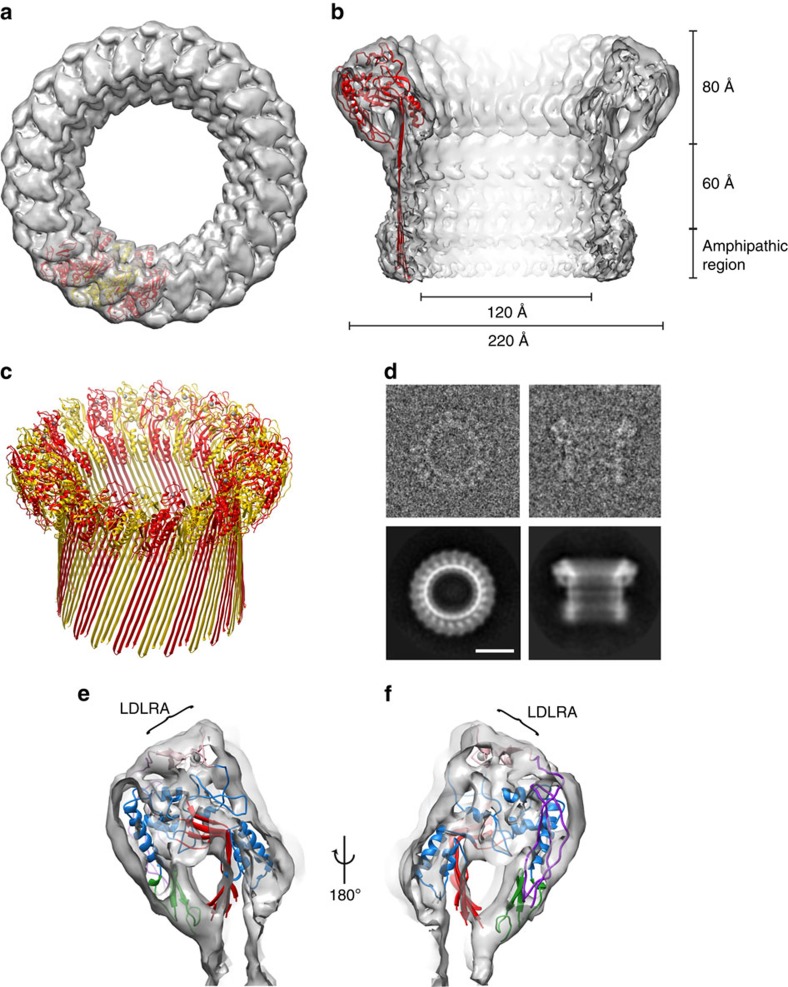
The structure of poly-C9. (**a**) Top–down view of a C9 trimer in the poly-C9 map and (**b**) cut through of the poly-C9 map with cartoon (red) of the poly-C9 model. Approximate dimensions and the predicted amphipathic region are indicated. (**c**) Cartoon of the full poly-C9 pore (alternating red and yellow monomers). The barrel is best modelled with the architecture *S=n/2* (ref. 42). (**d**) Cryo-EM end and side views of poly-C9 in individual images (top) and class averages (bottom). (**e**,**f**) With the exception of the mobile region of the MACPF domain (which in poly-C9 has rearranged in order to form the barrel), the crystal structure of C6 (PDB ID: 3T50) fits well into the map, with TMH1 and TMH2 omitted for clarity. In this figure the conserved β-sheet of the MACPF domain is in red, the body of the MACPF domain is in blue, the EGF domain in green, the TSP1 domain in purple and the LDLRA domain in pink (labelled).

To understand MAC assembly we determined the 8 Å single-particle cryo-EM reconstruction of soluble poly-C9 from 5,000 particles (Fig. 1a–d, Supplementary Figs 3–6). These data revealed a symmetrical assembly of 22 C9 monomers (Fig. 1a–c) that closely resembles the MAC^4^. The structure comprises a ring-shaped assembly of globular domains atop a large β-barrel (Fig. 1a,b). The latter part of the structure is flexible and is less well resolved than the top half of the structure. However, the diameter of the β-barrel (120 Å) is consistent with the predicted 88-stranded structure and is of sufficient size to permit passage of proteins such as lysozyme^15^. We further observed density, consistent with two N-glycosylation sites, one on each TMH sequence (Supplementary Figs 1 and 7). We observe a bulbous feature at the base of the β-barrel and suggest that this may be a consequence of structural rearrangements to protect the hydrophobic surface that ordinarily contacts the membrane (Fig. 1b). Higher resolution data will be required to validate this suggestion.

In the top, better-resolved portion of the map, the position of each of the four domains in C9 can be unambiguously assigned. Although no crystal structure of C9 is available, we were able to interpret the poly-C9 structure using the core TSP1-LDLRA-MACPF-EGF assembly from the crystal structure of C6 (refs 16, 17) (Fig. 1e,f; Supplementary Fig. 1). Indeed, only minor changes in domain orientation are required to dock the C6 structure into the bulk of the poly-C9 density (Fig. 1e,f).

### The TSP1 domain forms part of the oligomer interface

**Figure 2:**
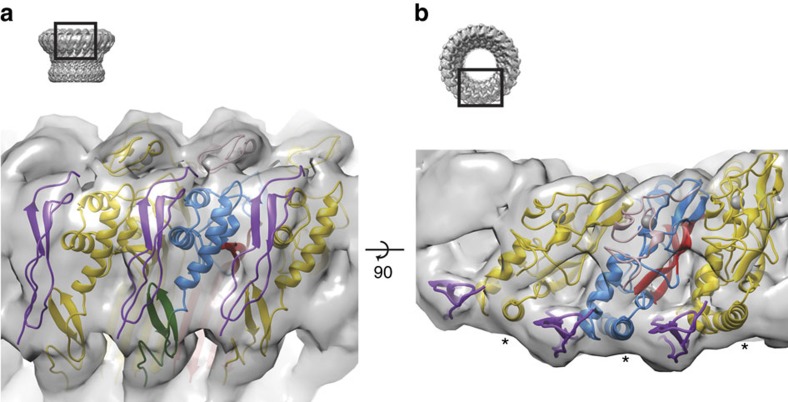
Interactions made by the TSP1 domain. (**a**) A view of the outside of the globular portion of the poly-C9 map showing the TSP1 domain (purple) located at each subunit interface. The central C9 monomer is coloured as in Fig. 1, with the monomers each side in dark yellow and purple (TSP1 domain). (**b**) A view from the top showing placement of the TSP1 domain between the C-terminal helix (marked with *) of each MACPF domain.

Structural studies on other MACPF/CDC proteins reveal that most interactions within the prepore or pore assembly appear to be formed between the relatively flat faces of the MACPF domain^8^,^11^,^13^. In contrast the poly-C9 structure reveals that the TSP1 domain packs against the C-terminal α-helix of the MACPF domain of an adjacent monomer and forms an additional and significant portion of the oligomer interface (Fig. 2). Thus in the pore form, each TSP1 domain is wedged between two C-terminal α-helices—one contributed in *trans* from an adjacent monomer and one in *cis*. This interaction at the outer edge of the ring-like assembly forms a quarter (∼690 Å^2^) of the total (∼3,000 Å^2^) surface buried in the globular, non-barrel region (Fig. 2). The remainder of the interacting surface is contributed by interactions between MACPF domains.

In the MAC it is anticipated that the MACPF domain of the related complement components C6, C7 and C8 form part of the overall circular assembly^3^. Like C9, C6–C8 all contain an analogous TSP1 domain that is functionally important (Supplementary Fig. 1)^14^. It is therefore suggested that the TSP1 domain of each protein in the complete MAC will be positioned at the subunit interface. Indeed, we suggest that the specialized TSP1/MACPF interactions likely explain the unusual ability of the nascent MAC to recruit components directly from solution. In contrast, proteins such as perforin, pleurotolysin and CDCs lack a TSP1 equivalent and do not readily self-assemble in solution. Instead, they require membrane anchoring via ancillary domains in order to oligomerise. Indeed, it is known from the study of receptors that restriction to the membrane plane can favour oligomerisation through weak protein–protein interactions^18^.

### Conformational transitions during pore formation

**Figure 3:**
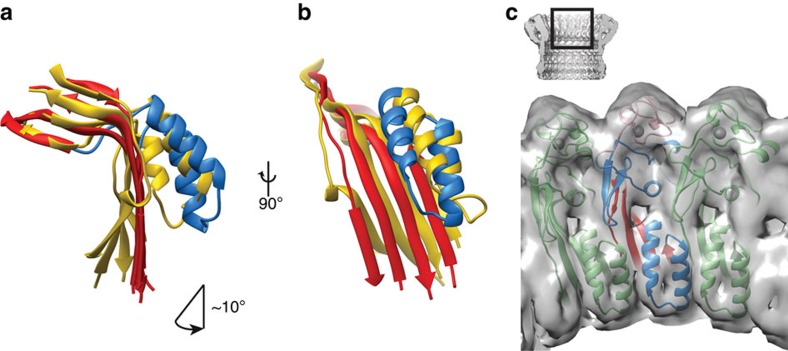
Comparison between the structure of C6 (PDB ID 3T5O; yellow) and model of poly-C9 (red/blue). The shift of the central bent β-sheet (red) shows (**a**) an ∼10° rotation of the bottom half of the sheet together with (**b**) an ∼5.5 Å lateral movement. (**c**) The HTH region (a pair of α-helices) lines the pore lumen. A trimer is shown with the central monomer coloured red, blue and pink.

We next examined the conformational changes that take place in the transition from the soluble monomer to the pore form. Comparison with C6 suggests that the largest conformational rearrangements during the transition from the monomer to the pore form take place within the MACPF domain^19^,^20^. The bottom half of the central β-sheet is rotated by ∼10 ° relative to its position in C6. This movement shifts the lower part of the β-sheet laterally by ∼5.5 Å (Fig. 3a,b). Concomitantly with this change, TMH1 and TMH2 unravel to form the β-barrel (Fig. 1b).

The lateral movement in the central sheet of the MACPF domain repositions the conserved helix-turn-helix (HTH) region that sits on top of TMH2 in the soluble monomeric form. Consistent with this, the top of the poly-C9 pore lumen is lined by pairs of α-helices (Fig. 3c). Previous mutagenesis and structural studies on the fungal MACPF protein pleurotolysin, as well as the CDC suilysin, suggest a role of the HTH region in pre-pore assembly and in controlling the transition to the pore^13^,^21^.

## Discussion

The structure of poly-C9 provides mechanistic insight into how components of the MAC may assemble through additional interactions mediated via the TSP1 domain. Furthermore, the structure provides insights into self-association by MACPF domain-containing proteins more generally. In particular, our present poly-C9 structure may resolve the controversy regarding the orientation of perforin in the pore assembly. Our previous analysis of the low-resolution EM structure of the perforin pore suggested that perforin monomers are orientated in the pore assembly opposite to the CDCs and pleurotolysin^8^,^11^. The latter two proteins, however, share very limited (<10%) sequence identity in the MACPF domain with perforin, whereas C9 is more closely related (∼25% identity). Accordingly, we superposed the perforin structure onto the poly-C9 model. This suggests that perforin most likely oligomerises similarly to C9, following minor rearrangements of the TMH2 and HTH domains (Supplementary Fig. 8). We note that residues shown through mutagenesis studies to interact at the pore interface are brought into close proximity with one another^22^. Further, the absence of the TSP1 domain in perforin at the outer edge of the pore assembly may explain the heterogeneity in perforin pore size and shape. We thus conclude that the present 8-Å-resolution poly-C9 map thus provides a better model for the perforin assembly.

Finally, the new structural insights may help explain how the MAC assembles with respect to target cell membranes. In the current view, C7 and C8 sequentially insert into the membrane, anchoring it in place before the recruitment of multiple copies of C9. However, this mechanism contrasts with the current view of the MACPF/CDC pore formation, in which the amphipathic hairpins are proposed to be inserted in a concerted fashion in the context of a complete or incomplete ring^11^,^23^. The latter mechanism seems more plausible because the conformational change in the MACPF domain during membrane insertion is extensive and would be predicted to disfavour the addition of new subunits. The poly-C9 structure provides new insights into this problem. The additional TSP1/MACPF interactions involve regions of the molecule that do not undergo significant conformational change. We therefore hypothesize that the TSP1-mediated interactions may permit addition of C8 and C9 to a nascent MAC that has already entered the target membrane.

To conclude, we have determined the structure of poly-C9 at a resolution sufficient to confidently position individual domains and to resolve helical features in density. Our data further reveal an unexpected contribution of domains ancillary to the MACPF domain that likely function to stabilize the overall assembly and the top half of the β-barrel pore.

## Methods

### Protein purification

Out-of-date apheresis human plasma was supplied by the Australian Red Cross and stored at −80 °C until required. This project was deemed by the Monash University Human Research Ethics Committee (project CF14/3761–2014001968) to be exempt from ethical review.

Plasma C9 was purified using protocols adapted from established protocols^24^,^25^. Briefly, apheresis plasma treated with 0.1 mM PMSF, 0.1 mM benzamidine, 0.5 mM EDTA and one protease inhibitor cocktail tablet per 100 ml plasma (Roche) was diluted with 0.4 volumes of ultrapure water at 4 °C. Protein was precipitated with 20% (w/v) PEG 4000 and re-suspended in 10 mM sodium phosphate pH 7.4, 45 mM NaCl, 10 mM EDTA. The suspension was passed over ID 2.5 cm × 4 cm loosely packed lysine resin (lysine sepharose 4b, GE Healthcare Life Sciences), and the flow through was then passed over ID 5 cm × 4 cm of DEAE resin (DEAE sepharose fast flow, GE Healthcare Life Sciences). The protein was eluted with a gradient from 10 mM sodium phosphate pH 7.4, 45 mM NaCl, 10 mM EDTA to 10 mM sodium phosphate pH 7.4, 350 mM NaCl, 10 mM EDTA. C9-containing fractions from DEAE were pooled and loaded onto ID 2.5 cm × 4 cm ceramic hydroxyapatite resin (CHT type I, BioRAD) equilibrated in 10 mM sodium phosphate pH 7.0, 100 mM NaCl. Protein was eluted with a sodium phosphate gradient from 45 to 350 mM, pH 8.1. The fractions containing C9 were further purified by size exclusion chromatography (Superdex 200 resin, GE Healthcare Life Sciences) in ID 2.6 cm × 60 cm column in 10 mM Hepes pH 7.2, 200 mM NaCl and 10 mM EDTA.

The protein underwent an additional chromatography step using MonoQ ion-exchange chromatography (GE Healthcare Life Sciences). Here, pooled fractions from the size exclusion chromatography step were concentrated using a 30 kDa MWCO centricon concentrator and buffer exchanged 2–3 times into 10 mM Tris-HCl pH 7.2, 100 mM NaCl. Buffer-exchanged protein was loaded onto a 1 ml MonoQ column and eluted over a linear gradient from: 10 mM Tris-HCl pH 7.2, 100 mM NaCl to 10 mM Tris-HCl pH 7.2, 350 mM NaCl. Purified protein that was shown to be haemolytically active and able to assemble into a complete MAC was used for EM experiments.

### Haemolytic assays and MAC assembly on ghost cell membranes

Sheep red blood cells (sRBC) were washed with DGHB^++^ pH 7.4 (Dextrose Gelatin HEPES Buffer; with 2.5% (w/v) D-glucose, 0.1% (w/v) gelatin, 5 mM HEPES pH 7.4, 71 mM NaCl, 0.15 mM CaCl_2_, 0.5 mM MgCl_2_,). In all, 6.5 × 10^8^ sRBC were sensitized with an equal volume of anti-sheep antibody to a concentration of 0.75 mg ml^−1^ and incubated at 30 °C for 30 min to activate the classical pathway. Excess antibody was washed off before reactions. The lysis reactions were set up in triplicate with 0.2 ml sRBC (3.75 × 10^6^ cells), 1 μl of C9-depleted serum (Complement Technology, USA) and 15 μl of C9 (initial concentration 8 μg ml^−1^) and twofold dilutions of purified C9 in thin-walled PCR tubes. Reaction tubes were incubated at 37 °C on a PCR heat block for 30 min and immediately placed at 4 °C, then centrifuged for 20 s. The supernatant (150 μl) of the lysis reactions was transferred to a 96-well plate and absorbance at 405 nm was measured. The reactions were normalized to 0% lysis with a buffer control or to a reaction containing PlyA and PlyB^13^ for 100% lysis.

Ghost membranes were prepared with rabbit red blood cells (rRBC) washed with DGHB^++^. Packed red blood cells were pre-incubated with C9-depleted serum for 15 min at 37 °C. The rRBCs were washed of excess sera and re-suspended in DGHB^++^. Purified C9 protein was added to rRBCs and incubated at 37 °C for 15 min. Reactions were immediately centrifuged for 30 s at 16,100 r.c.f. and the supernatant transferred to new tubes. The supernatant was further centrifuged for 10 min at 16,100 r.c.f. and the pellet containing membranes was washed once with DGHB^++^ and then resuspended in 10 mM phosphate buffer pH 8.0, 50 mM NaCl to make ghosts. Carbon-coated copper grids containing formvar were glow discharged, then floated over 8 μl of re-suspended ghosts followed by staining with 2% (w/v) uranyl acetate for 1 min. Pores were examined on a Hitachi H7500 TEM at 80 kV.

### Characterization of the glycosylation state

Purified C9 was reduced with 2 mM DTT, alkylated with 5 mM iodoacetamide and digested with trypsin (Promega) in 1:40 ratio at 37 °C overnight. The digest was desalted with C18-packed tips (OMIX, Agilent) before nanoLC-MS/MS (Dionex Ultimate 3000 LC coupled to QExactive Plus, Thermo). Peptides (∼1 μg) were loaded on a 2 cm trap column (100 μm ID, Acclaim PepMap 100, Thermo Scientific) in 2% (v/v) acetonitrile, 0.1% (v/v) trifluoroacetic acid and resolved on a 50 cm column (75 μm ID, Acclaim PepMapRSLC, Thermo Scientific) with a non-linear 25 min gradient from 2% (v/v) to 34% (v/v) acetonitrile in 0.1% (v/v) formic acid. Spectra were acquired in a Top12 strategy with full scans (375–1,800 m z^−1^) acquired at 70,000 resolution and data-dependent HCD MS2 spectra acquired at 17,500 resolution. Peptide assignment was performed with the Preview and Byonic software (Protein Metrics^26^) utilizing Preview-determined modifications and mass tolerances, a focused human database from an initial Byonic search and N- and O-glycosylation databases for assignment of glycan compositions. All glycan composition assignments were manually validated. Skyline software (University of Washington) was used for semi-quantitative assessment of site-specific glycan compositions, using parent scan extracted ion chromatograms^27^ (Supplementary Fig. 7).

### Cryo-EM sample preparation and data acquisition

Monomeric C9 was polymerized by overnight incubation at 1 mg ml^−1^ and 37 °C. The resulting poly-C9 was applied to lacey carbon-coated copper grids (Agar, UK) and frozen with a FEI Vitrobot Mark III (FEI, Eindhoven) at 22 °C and 100% humidity. Images were recorded manually on a Tecnai G2 Polara microscope (FEI) operating at 300 kV with a Quantum energy filter and K2 Summit detector (Gatan, UK) in counting mode, at a pixel size of 2.76 Å. Exposures were recorded at 1.2 electrons (Å^2^)^−1^ s^−1^ for 25 s, with defocus values ranging from 1.2 to 4.9 μm (Supplementary Fig. 3).

### 3D reconstruction of poly-C9

The detector movies were aligned using IMOD^28^. CTF parameters were determined with CTFFIND4 (ref. 29). A total of 10,800 particles were extracted manually using Boxer (EMAN 1.9) (ref. 30). Classification and refinement were performed using RELION^31^. 2D classification in IMAGIC^29^ revealed mainly end views with 22-fold symmetry, with a small fraction of particles having 21- or 23-fold symmetry (Supplementary Fig. 3). The initial model with 22-fold symmetry was created by angular reconstitution from 2D class averages of particles with all orientations in IMAGIC^32^ and refined by projection matching using SPIDER^33^. A subset of ∼5,000 particles in side and tilted views (homogeneous with respect to diameter of the wide part of the ring, corresponding to the 22-mers) was refined with RELION using the initial model from SPIDER filtered to 20 Å. Twenty-two-fold symmetry was applied during refinement. The final map was corrected to the modulation transfer function of the detector and sharpened by applying a B-factor^34^ determined by RELION. The final resolution calculation based on gold-standard FSC was estimated at 0.5 and 0.143 FSC in RELION. Local resolution was estimated using the ResMap program^35^ (Supplementary Fig. 4).

### Determination of handedness

In order to determine the absolute hand of the 3D reconstruction, the crystallographic structure of C6 was fitted into the map as well as into the map with opposite handedness. Although the fit of the C6 conformation was found to favour one hand over the other, the differences in cross-correlations were too small to conclusively assign the hand of the map (C6: 0.63 versus 0.59; calculated using the Chimera software^36^).

To resolve this issue, we examined the fit to both maps of the conserved structural features of the MACPF domain. The C-terminal α-helical bundle of the MACPF domain (Supplementary Fig. 5) is likely to be clearly discernable in an 8-Å-resolution cryo-EM density. Its characteristic arrangement of α-helices is asymmetric and highly conserved in all the crystallographic structures of MAC components^17^. We therefore expected that it should be possible to identify the correct hand from analysis of the fit of this structural motif in the enantiomeric maps (Supplementary Fig. 5b,c).

Accordingly, we found that the map in Supplementary Fig. 5b showed distinct density corresponding to the C-terminal α-helical bundle. The region of the map identified by rigid body fitting excellently reproduces the topology and length of the α-helices. Conversely, the map in Supplementary Fig. 5c produces a comparatively poor agreement with the fitted position of C6 (Supplementary Fig. 5c). We concluded that the map in Supplementary Fig. 5b represents the correct hand.

### Fitting of atomic models

A homology model of C9 was fitted into the EM map by using a combination of manual, rigid body and flexible fitting. The C9 homology model was generated using the crystallographic structures of C6 (PDB IDs: 3T5O, 4A5W) and C8 (2RD7, 3OJYA, 3OJYB) and Modeller 9.14 (ref. 37). The TMH1/2 regions were discarded because these regions form a β-barrel in poly-C9.

Five symmetry-related monomers were then subjected to flexible fitting (MDFF methodology as implemented in NAMD 2.10 (ref. 38) using symmetry restraints^39^. The protein secondary structure was restrained to avoid overfitting. Oligomeric main chain hydrogen bonds between the β-sheets forming the top of the β-barrel were also restrained to reproduce the pattern conserved in the MACPF/CDC superfamily^13^,^40^. Two independent 5-ns simulations were performed in vacuo at 310 K (γ=0.3; 1 fs time step; 12 Å cutoff for long-range interaction) using the CHARMM36 force field^41^ and followed by 5,000 steps of energy minimization (γ=0.5). The resulting model with the highest CC (0.93; Molprobity score of 1.15) was replicated with C22 symmetry and combined with a structural model of the 88-stranded β-barrel (architecture S=n/2 (ref. 42) using Modeller, thus extending the β-strands of the central MACPF β-sheet as performed in Leung *et al*.^11^ Lukoyanova *et al*.^13^ and Reboul *et al*.^43^ The final poly-C9 22-mer model (CC of 0.94) is shown in Fig. 1.

In order to assess the reliability of the fitting procedure, the flexible fitting step was repeated using cryo-EM maps calculated from randomly partitioned half-sets, independently refined using RELION and used to determine the resolution of the final cryo-EM map (see EM methods). Individual residue RMSDs of both fitted models were calculated with respect to the structural model obtained from the whole data set (Supplementary Fig. 6). Structural elements displaying an overall high RMSD (for example, not fitted in a reproducible manner; >3.5 Å) were not included in the final structural model.

## Additional information

**How to cite this article:** Dudkina, N. V. *et al*. Structure of the poly-C9 component of the complement membrane attack complex. *Nat. Commun.* 7:10588 doi: 10.1038/ncomms10588 (2016).

## Supplementary Material

Supplementary Information Supplementary Figures 1-8 and Supplementary References

## References

[b1] Kaufmann S. H. E. Immunology's foundation: the 100-year anniversary of the Nobel Prize to Paul Ehrlich and Elie Metchnikoff. Nat. Immunol. 9, 705–712 (2008). 18563076 10.1038/ni0708-705

[b2] Walport M. J. Advances in immunology: complement (first of two parts). N. Engl. J. Med. 344, 1058–1066 (2001). 11287977 10.1056/NEJM200104053441406

[b3] Podack E. R. Molecular mechanisms of cytolysis by complement and by cytolytic lymphocytes. J. Cell Biochem. 30, 133–170 (1986). 2422185 10.1002/jcb.240300205

[b4] Tschopp J., Podack E. R. & Müller-Eberhard H. J. Ultrastructure of the membrane attack complex of complement: detection of the tetramolecular C9-polymerizing complex C5b-8. Proc. Natl Acad. Sci. USA 79, 7474–7478 (1982). 6961424 10.1073/pnas.79.23.7474 PMC347362

[b5] Tschopp J., Muller-Eberhard H. J. & Podack E. R. Formation of transmembrane tubules by spontaneous polymerization of the hydrophilic complement protein C9. Nature 298, 534–538 (1982). 7099251 10.1038/298534a0

[b6] Rosado C. J. . A common fold mediates vertebrate defense and bacterial attack. Science 317, 1548–1551 (2007). 17717151 10.1126/science.1144706

[b7] Hadders M. A., Beringer D. X. & Gros P. Structure of C8alpha-MACPF reveals mechanism of membrane attack in complement immune defense. Science 317, 1552–1554 (2007). 17872444 10.1126/science.1147103

[b8] Tilley S. J., Orlova E. V., Gilbert R. J. C., Andrew P. W. & Saibil H. R. Structural basis of pore formation by the bacterial toxin pneumolysin. Cell 121, 247–256 (2005). 15851031 10.1016/j.cell.2005.02.033

[b9] Shepard L. A. . Identification of a membrane-spanning domain of the thiol-activated pore-forming toxin *Clostridium perfringens* perfringolysin O: An alpha-helical to beta-sheet transition identified by fluorescence spectroscopy. Biochemistry 37, 14563–14574 (1998). 9772185 10.1021/bi981452f

[b10] Czajkowsky D. M., Hotze E. M., Shao Z. & Tweten R. K. Vertical collapse of a cytolysin prepore moves its transmembrane beta-hairpins to the membrane. EMBO J. 23, 3206–3215 (2004). 15297878 10.1038/sj.emboj.7600350 PMC514522

[b11] Leung C. . Stepwise visualization of membrane pore formation by suilysin, a bacterial cholesterol-dependent cytolysin. Elife 3, e04247 (2014). 25457051 10.7554/eLife.04247 PMC4381977

[b12] Law R. H. P. . The structural basis for membrane binding and pore formation by lymphocyte perforin. Nature 468, 447–451 (2010). 21037563 10.1038/nature09518

[b13] Lukoyanova N. . Conformational changes during pore formation by the perforin-related protein pleurotolysin. PLoS Biol. 13, e1002049 (2015). 25654333 10.1371/journal.pbio.1002049 PMC4318580

[b14] Scibek J. J., Plumb M. E. & Sodetz J. M. Binding of human complement C8 to C9: role of the N-terminal modules in the C8 alpha subunit. Biochemistry 41, 14546–14551 (2002). 12463754 10.1021/bi026641j

[b15] Martinez R. J. & Carroll S. F. Sequential metabolic expressions of the lethal process in human serum-treated *Escherichia coli*: role of lysozyme. Infect. Immun. 28, 735–745 (1980). 6156906 10.1128/iai.28.3.735-745.1980 PMC551012

[b16] Hadders M. A. . Assembly and regulation of the membrane attack complex based on structures of C5b6 and sC5b9. Cell Rep. 1, 200–207 (2012). 22832194 10.1016/j.celrep.2012.02.003 PMC3314296

[b17] Aleshin A. E. . Structure of complement C6 suggests a mechanism for initiation and unidirectional, sequential assembly of membrane attack complex (MAC). J. Biol. Chem. 287, 10210–10222 (2012). 22267737 10.1074/jbc.M111.327809 PMC3323040

[b18] Wu Y., Vendome J., Shapiro L., Ben-Shaul A. & Honig B. Transforming binding affinities from three dimensions to two with application to cadherin clustering. Nature 475, 510–513 (2011). 21796210 10.1038/nature10183 PMC3167384

[b19] DiScipio R. G. The size, shape and stability of complement component C9. Mol. Immunol. 30, 1097–1106 (1993). 8366860 10.1016/0161-5890(93)90156-6

[b20] DiScipio R. G. & Berlin C. The architectural transition of human complement component C9 to poly(C9). Mol. Immunol. 36, 575–585 (1999). 10499811 10.1016/s0161-5890(99)00073-5

[b21] Ramachandran R., Tweten R. K. & Johnson A. E. Membrane-dependent conformational changes initiate cholesterol-dependent cytolysin oligomerization and intersubunit beta-strand alignment. Nat. Struct. Mol. Biol. 11, 697–705 (2004). 15235590 10.1038/nsmb793

[b22] Baran K. . The molecular basis for perforin oligomerization and transmembrane pore assembly. Immunity 30, 684–695 (2009). 19446473 10.1016/j.immuni.2009.03.016

[b23] Sonnen A. F.-P., Plitzko J. M. & Gilbert R. J. C. Incomplete pneumolysin oligomers form membrane pores. Open Biol. 4, 140044 (2014). 24759615 10.1098/rsob.140044 PMC4043118

[b24] Biesecker G. & Müller-Eberhard H. J. The ninth component of human complement: purification and physicochemical characterization. J. Immunol. 124, 1291–1296 (1980). 6766971

[b25] Biesecker G., Lachmann P. & Henderson R. Structure of complement poly-C9 determined in projection by cryo-electron microscopy and single particle analysis. Mol. Immunol. 30, 1369–1382 (1993). 8232323 10.1016/0161-5890(93)90098-v

[b26] Bern M., Kil Y. J. & Becker C. Byonic: advanced peptide and protein identification software. Curr. Protoc. Bioinformatics 40, 13.20.1–13.20.14 (2012). 10.1002/0471250953.bi1320s40 PMC3545648 23255153

[b27] Schilling B. . Platform-independent and label-free quantitation of proteomic data using MS1 extracted ion chromatograms in skyline: application to protein acetylation and phosphorylation. Mol. Cell Proteomics 11, 202–214 (2012). 22454539 10.1074/mcp.M112.017707 PMC3418851

[b28] Kremer J. R., Mastronarde D. N. & McIntosh J. R. Computer visualization of three-dimensional image data using IMOD. J. Struct. Biol. 116, 71–76 (1996). 8742726 10.1006/jsbi.1996.0013

[b29] Rohou A. & Grigorieff N. CTFFIND4: fast and accurate defocus estimation from electron micrographs. J. Struct. Biol. 192, 216–221 (2015). 26278980 10.1016/j.jsb.2015.08.008 PMC6760662

[b30] Ludtke S. J., Baldwin P. R. & Chiu W. EMAN: semiautomated software for high-resolution single-particle reconstructions. J. Struct. Biol. 128, 82–97 (1999). 10600563 10.1006/jsbi.1999.4174

[b31] Scheres S. H. W. RELION: implementation of a Bayesian approach to cryo-EM structure determination. J. Struct. Biol. 180, 519–530 (2012). 23000701 10.1016/j.jsb.2012.09.006 PMC3690530

[b32] van Heel M., Harauz G., Orlova E. V., Schmidt R. & Schatz M. A new generation of the IMAGIC image processing system. J. Struct. Biol. 116, 17–24 (1996). 8742718 10.1006/jsbi.1996.0004

[b33] Frank J. . SPIDER and WEB: processing and visualization of images in 3D electron microscopy and related fields. J. Struct. Biol. 116, 190–199 (1996). 8742743 10.1006/jsbi.1996.0030

[b34] Rosenthal P. B. & Henderson R. Optimal determination of particle orientation, absolute hand, and contrast loss in single-particle electron cryomicroscopy. J. Mol. Biol. 333, 721–745 (2003). 14568533 10.1016/j.jmb.2003.07.013

[b35] Kucukelbir A., Sigworth F. J. & Tagare H. D. Quantifying the local resolution of cryo-EM density maps. Nat. Methods 11, 63–65 (2014). 24213166 10.1038/nmeth.2727 PMC3903095

[b36] Pettersen E. F. . UCSF chimera—a visualization system for exploratory research and analysis. J. Comput. Chem. 25, 1605–1612 (2004). 15264254 10.1002/jcc.20084

[b37] Eswar N. . Comparative protein structure modeling using MODELLER. Curr. Protoc. Protein Sci. 50, 2.9.1–2.9.31 (2007). 10.1002/0471140864.ps0209s50 18429317

[b38] Trabuco L. G., Villa E., Mitra K., Frank J. & Schulten K. Flexible fitting of atomic structures into electron microscopy maps using molecular dynamics. Structure 16, 673–683 (2008). 18462672 10.1016/j.str.2008.03.005 PMC2430731

[b39] Chan K.-Y. . Symmetry-restrained flexible fitting for symmetric EM maps. Structure 19, 1211–1218 (2011). 21893283 10.1016/j.str.2011.07.017 PMC3412758

[b40] Sato T. K., Tweten R. K. & Johnson A. E. Disulfide-bond scanning reveals assembly state and β-strand tilt angle of the PFO β-barrel. Nat. Chem. Biol. 9, 383–389 (2013). 23563525 10.1038/nchembio.1228 PMC3661704

[b41] Huang J. & MacKerell A. D. CHARMM36 all-atom additive protein force field: validation based on comparison to NMR data. J. Comput. Chem. 34, 2135–2145 (2013). 23832629 10.1002/jcc.23354 PMC3800559

[b42] Reboul C. F., Mahmood K., Whisstock J. C. & Dunstone M. A. Predicting giant transmembrane β-barrel architecture. Bioinformatics 28, 1299–1302 (2012). 22467914 10.1093/bioinformatics/bts152

[b43] Reboul C. F., Whisstock J. C. & Dunstone M. A. A new model for pore formation by cholesterol-dependent cytolysins. PLoS Comput. Biol. 10, e1003791 (2014). 25144725 10.1371/journal.pcbi.1003791 PMC4140638

