# Supplementary material for: Structure of the poly-C9 component of the complement membrane attack complex
Source: Nat Commun. 2016 Feb 4;7:10588. doi: 10.1038/ncomms10588 (PMC4742998; doi:10.1038/ncomms10588)
Supplement: Supplementary Information — Supplementary Figures 1-8 and Supplementary References [file ncomms10588-s1.pdf]

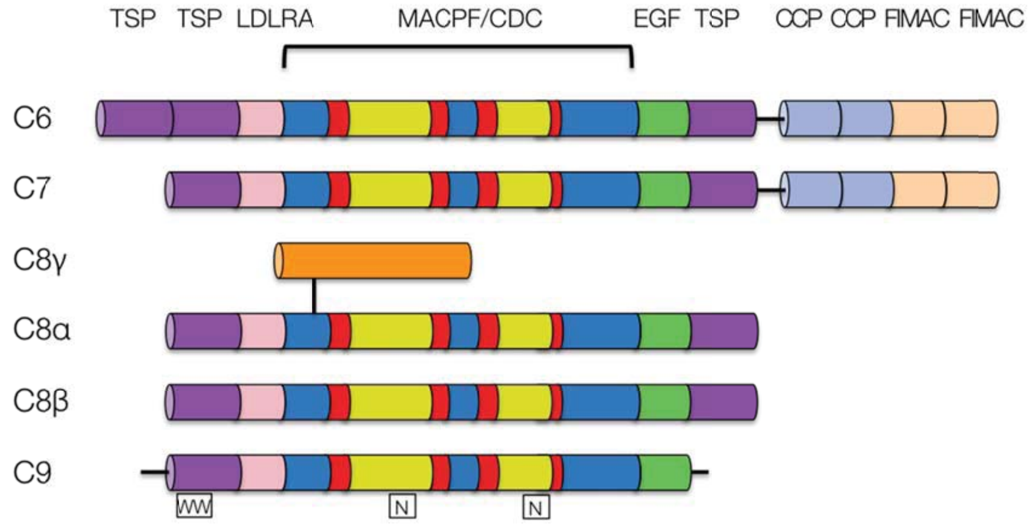

Supplementary Fig. 1. Schematic of domain composition of the Membrane Attack Complex proteins

Schematic of the domain composition of C9 in comparison with C6, C7, C8α, C8β. The schema of C9 includes the sites of C-mannosylation (WW) and N-glycosylation (N) of C9 as determined according to methods and in agreement with published data [1,2]. The two predicted TMH regions are also labelled. Colours are the same as used throughout the domain coloured figures. TSP = Thrombospondin Type 1 domain, LDLRA = Low-Density Lipoprotein Receptor Type A, MACPF = Membrane Attack Complex/Perforin / Cholesterol Dependent Cytolysin, EGF = Epidermal Growth Factor-like, CCP = Complement Control Protein, FIMAC = Factor I / Membrane Attack Complex domain. Colours are the same as used in Figs 1, 2 and 3.

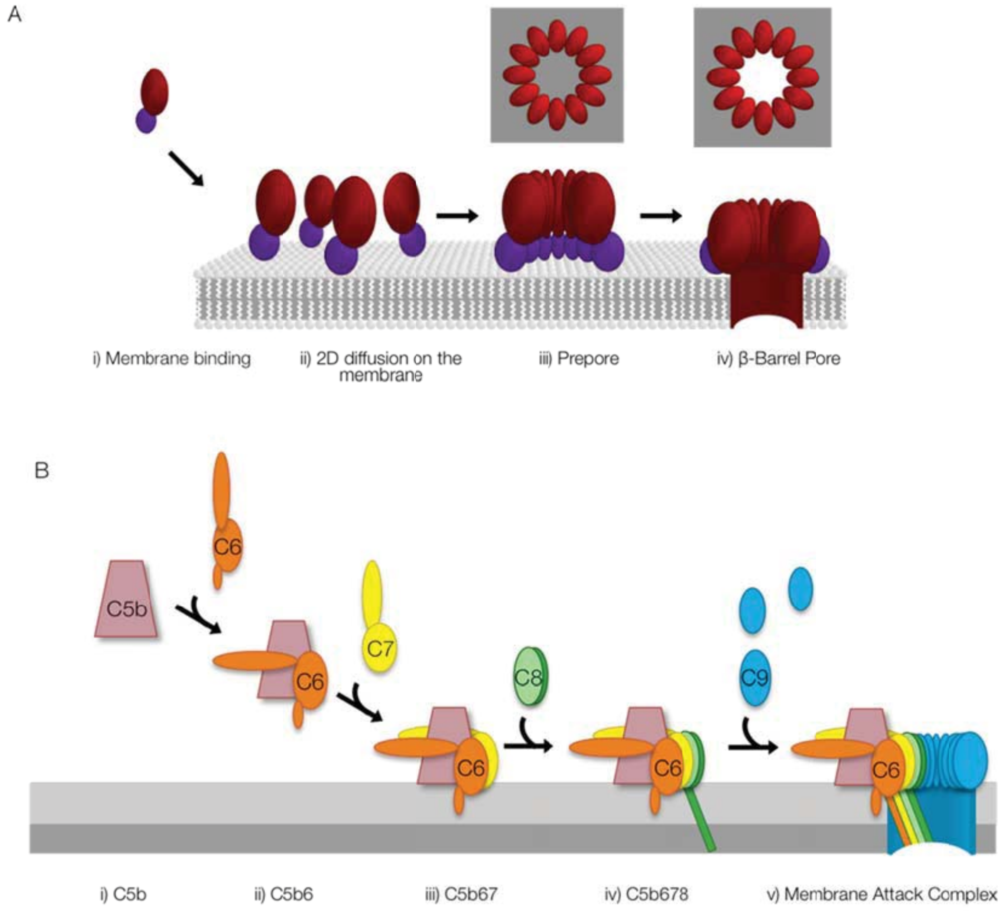

Supplementary Fig. 2 The general mechanism of MACPF/CDC pore forming proteins (adapted from [3]).

a) The CDC pore forming mechanism. i) Membrane recognition and binding, ii) two dimensional diffusion of the monomers on the membrane and iii) oligomerisation into the prepore state. iv) β-barrel pore formation, postulated to occur as a concerted, simultaneous insertion of the two TMH regions. Insets at the top show the state of the membrane in the prepore and pore states.

b) The general mechanism of the Membrane Attack Complex (MAC) formation. After formation of C5b (pink, panel (i)) there is sequential binding of C6 (orange, panel (ii)), C7 (yellow(iii)) and the C8 heterotrimer (green, C8γ not shown, panel (iv)). It is postulated that the C8α component (dark green) of C8 inserts its TMH2 region into the membrane at this stage. In panel (v) the final pore formation is depicted with the sequential addition of ~18 C9 molecules (blue; initial interface proposed to be with C8).

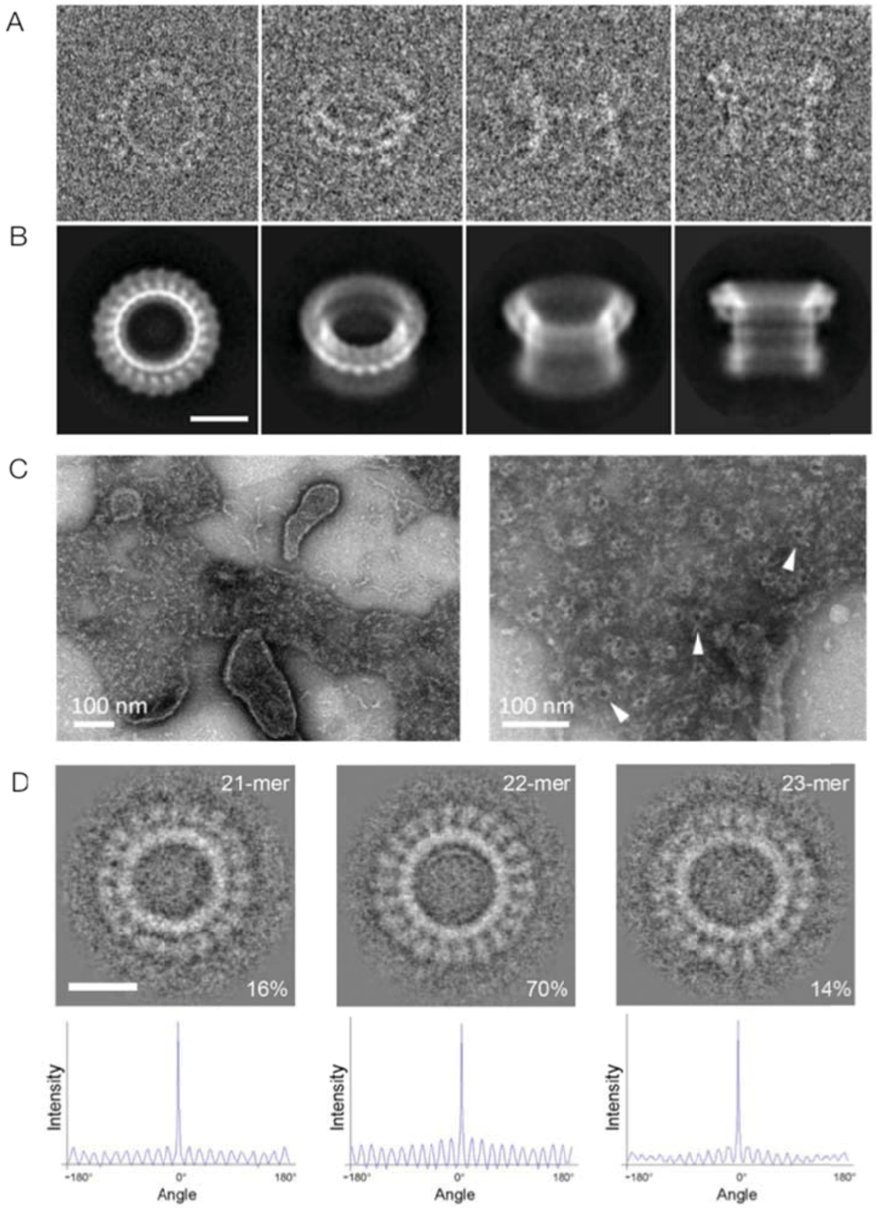

Supplementary Fig. 3 Electron microscopy of polyC9 and MAC.

Representative cryo-EM **(a)** views of four raw images of polyC9 with the **(b)** corresponding averaged views. Scale bar, 10 nm.

(c) Rabbit red blood cell ghosts incubated with C9 depleted serum without the addition of purified C9 (left) and with the addition of purified C9 (right). MAC pores are indicated by white arrows. **(d)** Symmetry of polyC9 pores. Representative averaged views of 21, 22 and 23-fold symmetric pores (upper row) with proportion of particles with each symmetry detected in the data set and corresponding rotational autocorrelations (lower row). The bar is 10 nm.

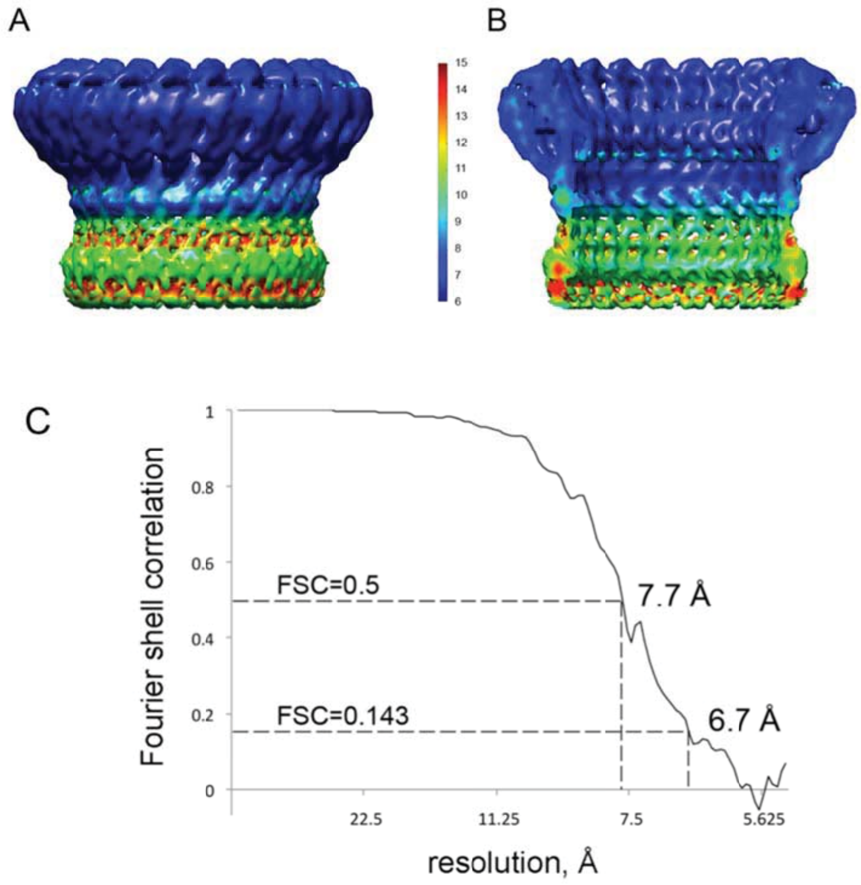

Supplementary Fig. 4 Map quality of poly-C9.

(a) A surface view of the sharpened final map colored according to local resolution. **(b)** As in A, but a cut-through view. **(c)** Fourier-shell correlation curve.

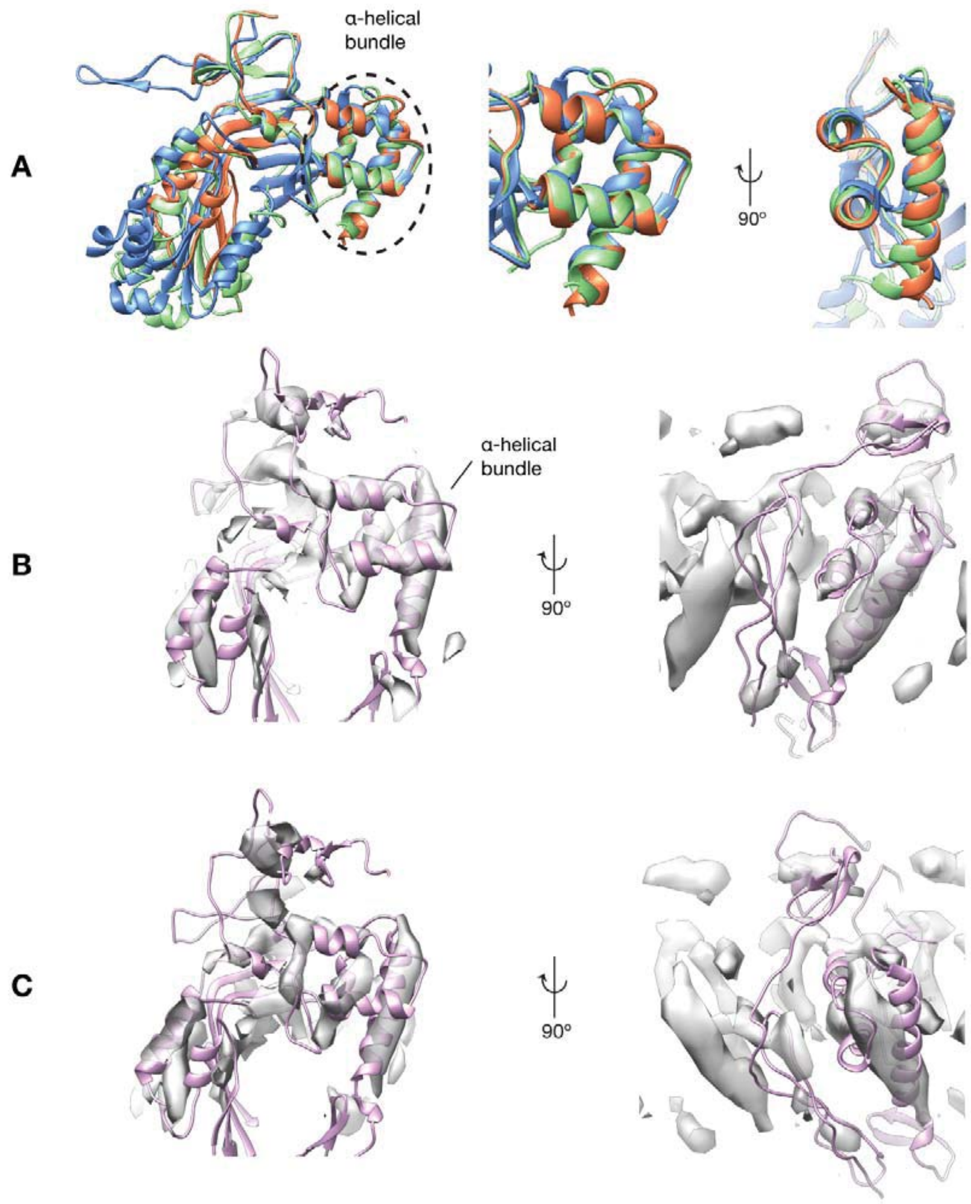

Supplementary Fig. 5 Structural alignment of the MACPF domain of MAC components and determination of handedness.

(a) The alignment identifies two crossed pairs of α-helices (far left, circled) as a highly structurally conserved region of the MACPF domain. The topology and lengths of the individual α-helices are identical in MAC components (middle and right panels). Superpositions of the crystallographic structures of C6 (PDB ID 3T5O, orange), C8α (PDB ID: 2RD7, green) and C8β (PDB ID: 3OJY chain B, blue). **(b)** The hand of map chosen in this study and **(c)** the mirrored map. The crystallographic conformation of C6 (PDB ID: 3T5O, pink) is in cartoon representation.

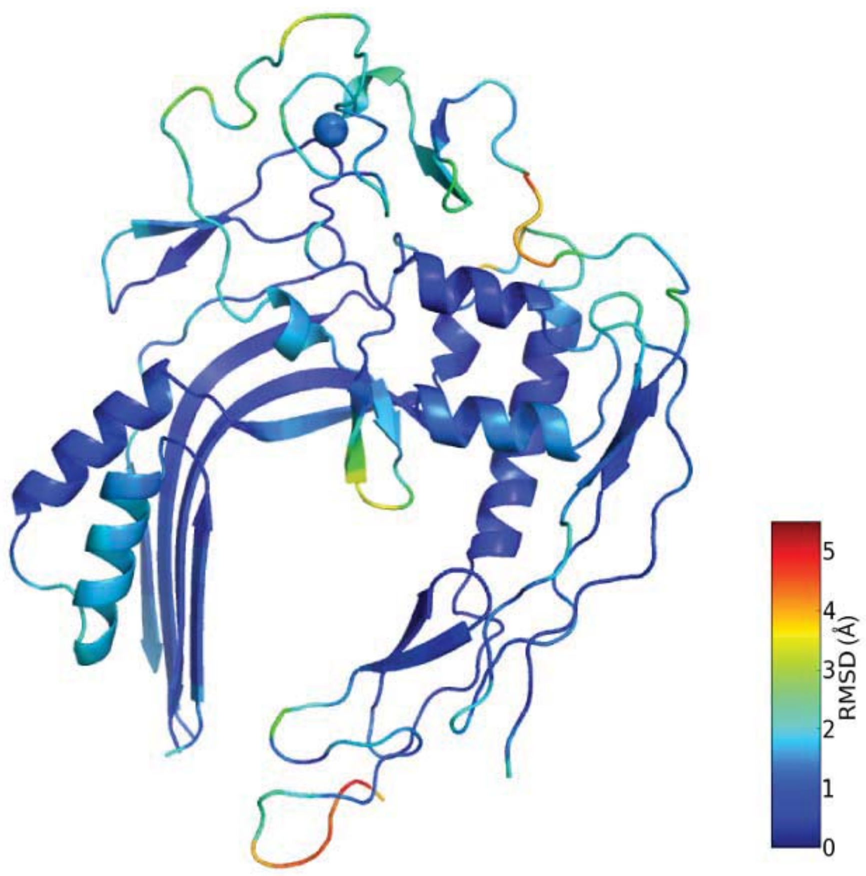

Supplementary Fig. 6 Reproducibility of the flexible fitting step.

Regions displaying a high RMSD are limited to the C-terminus and the loop at positions 74-79.

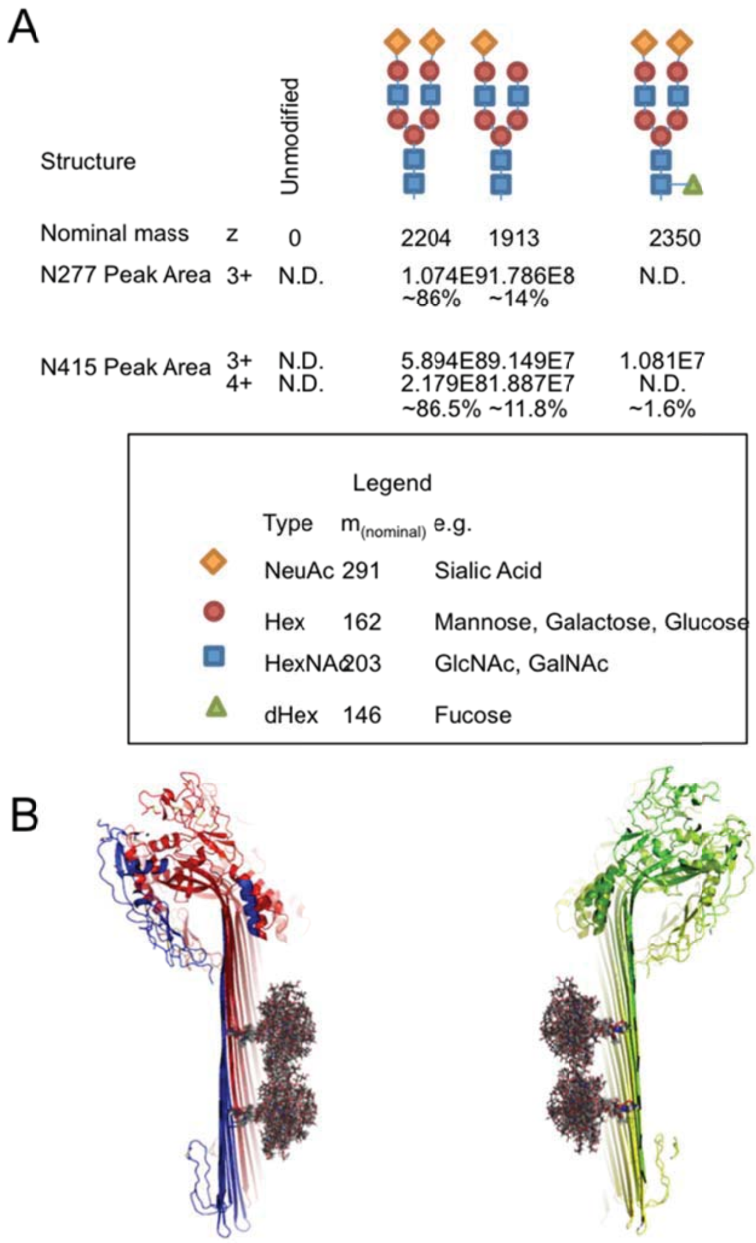

Supplementary Fig. 7 The N-glycosylation of C9

(a) MS-MS characterisation of the two N-glycan sites. Most of the N-glycans have two sialic acid groups each.

(b) Superposition of the glycan models from the NMR structure of human chorionic gonadotropin (PDB ID: 1HD4). These N-glycans lack the sialic groups but show the potential degrees of freedom of each of the 44 glycan groups located in the pore lumen.

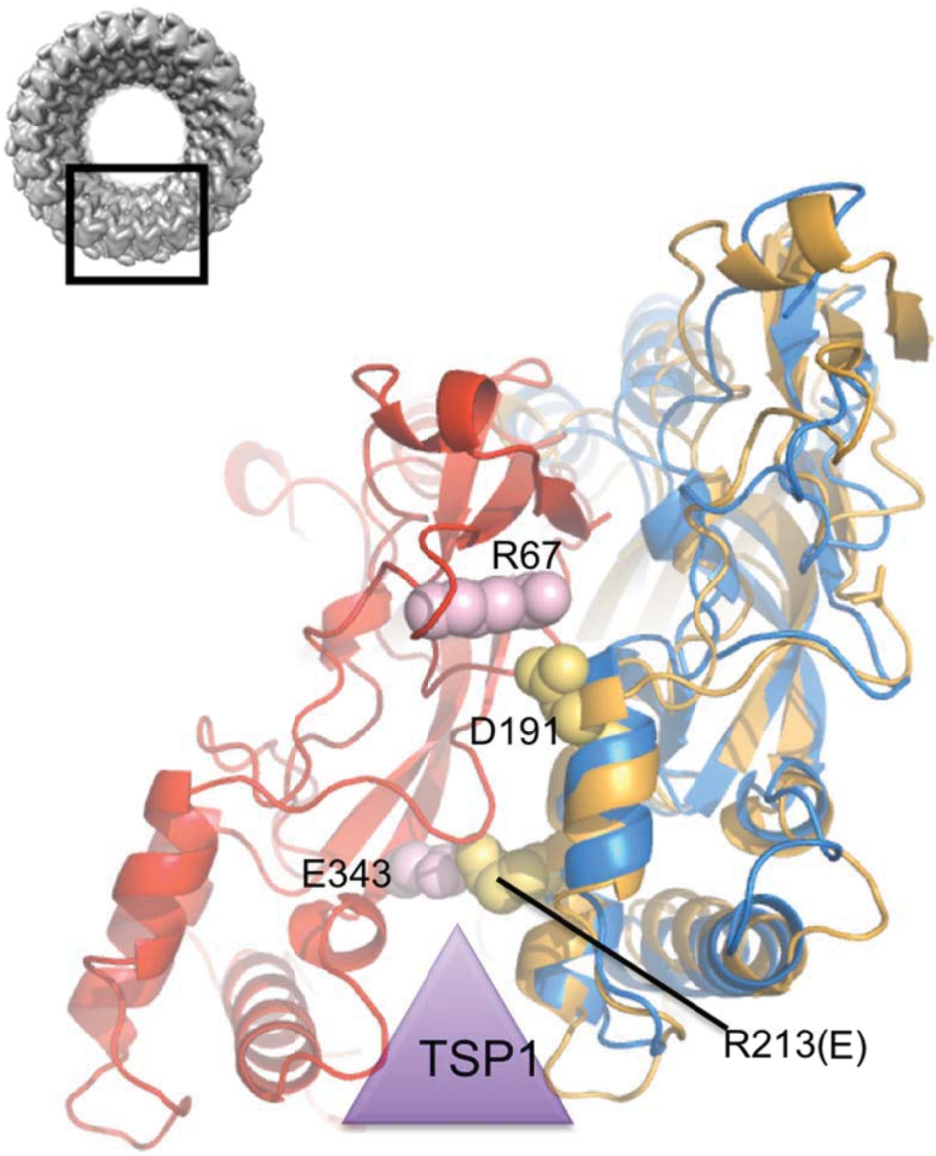

Supplementary Fig. 8 Superposition of perforin on the model of polyC9.

Illustration of the arrangement of two perforin molecules (red and orange) generated by superposition of the perforin structure (PDB ID: 3NSJ) on the model of poly-C9 (blue). In the superposition R213 (mutated to a Glu in 3NSJ) is in close proximity to E343 as previously predicted [4]. These data further suggest that D191 which has been shown to be important for pore formation forms a salt bridge with R67.

Supplementary References

1. Hofsteenge, J., Blommers, M., Hess, D., Furmanek, A. & Miroshnichenko, O. The four terminal components of the complement system are C-mannosylated on multiple tryptophan residues. *J. Biol. Chem.* **274,** 32786–94 (1999).

2. DiScipio, R. G. & Hugli, T. E. The architecture of complement component C9 and poly(C9). *J. Biol. Chem.* **260,** 14802–9 (1985).

3. Kondos, S. C. *et al.* The structure and function of mammalian membrane-attack complex/perforin-like proteins. *Tissue Antigens* **76,** 341–51 (2010).

4. Baran, K. *et al.* The molecular basis for perforin oligomerization and transmembrane pore assembly. *Immunity* **30,** 684–95 (2009).
